# Improving the safety of cell therapy with the TK-suicide gene

**DOI:** 10.3389/fphar.2015.00095

**Published:** 2015-05-05

**Authors:** Raffaella Greco, Giacomo Oliveira, Maria Teresa Lupo Stanghellini, Luca Vago, Attilio Bondanza, Jacopo Peccatori, Nicoletta Cieri, Sarah Marktel, Sara Mastaglio, Claudio Bordignon, Chiara Bonini, Fabio Ciceri

**Affiliations:** ^1^Unit of Hematology and Bone Marrow Transplantation, Division of Regenerative Medicine, Stem Cells and Gene Therapy, IRCCS San Raffaele Scientific Institute, MilanItaly; ^2^Experimental Hematology Unit, Division of Immunology, Transplantation and Infectious Diseases, Program in Immunology and Bio-immunotherapy of Cancer, IRCCS San Raffaele Scientific Institute, MilanItaly; ^3^Unit of Molecular and Functional Immunogenetics, Division of Regenerative Medicine, Stem Cells and Gene Therapy, IRCCS San Raffaele Scientific Institute, MilanItaly; ^4^Leukemia Immunotherapy Unit, Immunology, Transplantation and Infectious Diseases, IRCCS San Raffaele Scientific Institute, MilanItaly; ^5^Vita-Salute San Raffaele University, MolMed S.p.A., MilanItaly

**Keywords:** cellular adoptive immunotherapy, gene therapy, allogeneic hematopoietic stem cell transplantation, suicide gene therapy, TK cells

## Abstract

While opening new frontiers for the cure of malignant and non-malignant diseases, the increasing use of cell therapy poses also several new challenges related to the safety of a living drug. The most effective and consolidated cell therapy approach is allogeneic hematopoietic stem cell transplantation (HSCT), the only cure for several patients with high-risk hematological malignancies. The potential of allogeneic HSCT is strictly dependent on the donor immune system, particularly on alloreactive T lymphocytes, that promote the beneficial graft-versus-tumor effect (GvT), but may also trigger the detrimental graft-versus-host-disease (GvHD). Gene transfer technologies allow to manipulate donor T-cells to enforce GvT and foster immune reconstitution, while avoiding or controlling GvHD. The suicide gene approach is based on the transfer of a suicide gene into donor lymphocytes, for a safe infusion of a wide T-cell repertoire, that might be selectively controlled *in vivo* in case of GvHD. The herpes simplex virus thymidine kinase (HSV-TK) is the suicide gene most extensively tested in humans. Expression of HSV-TK in donor lymphocytes confers lethal sensitivity to the anti-herpes drug, ganciclovir. Progressive improvements in suicide genes, vector technology and transduction protocols have allowed to overcome the toxicity of GvHD while preserving the antitumor efficacy of allogeneic HSCT. Several phase I-II clinical trials in the last 20 years document the safety and the efficacy of HSV-TK approach, able to maintain its clear value over the last decades, in the rapidly progressing horizon of cancer cellular therapy.

## Introduction

Cellular therapy is an emerging therapeutic modality, designed to treat cancer, genetic and autoimmune diseases, currently raising high enthusiasm. Cancer immunotherapy in particular has been selected as the major breakthrough of 2013 (Couzin-Frankel, 2013), supporting new approaches that will bring this strategy into wide clinical development in a very near future.

The major challenge for immunotherapy is to translate advances in cellular and molecular immunology into strategies that effectively and safely enhance clinical responses in cancer patients. This aim has been pursued by different strategies that include non-specific immunomodulation approaches based on the administration of cytokines such as IL-2 (Rosenberg et al., 1998a), or more recently based on the blockade of inhibitory signals, such as CTLA4 or the PD1/PDL1 axes (Hodi et al., 2010; Prosser et al., 2012). The active immunization of patients against their metastatic cancer, using the so-called “cancer vaccines,” represents another immunotherapeutic approach (Rosenberg et al., 1998b; Brahmer et al., 2010). The expanding knowledge on the field of cancer biology has also enabled passive immunotherapeutic approaches, such as antibody-mediated therapy (Weiner et al., 2010) and adoptive cellular therapy (ACT), involving the *ex vivo* identification of autologous or allogeneic lymphocytes with anti-tumor activity (June, 2007a,b). Targeting destruction of malignancies by enhancing T-cell responses is an attractive therapeutic modality since it potentially combines excellent specificity with potent anti-tumor activity. However, ACT has been limited, until recently, by several restrictions, including the low frequency of naturally occurring tumor-specific T-cells displaying proper anti-tumor avidity, the low potency of the biotechnological tools employed, and the rapid ensuing of T-cell exhaustion or tumor immune escape (Pardoll, 2012).

The most effective and consolidated adoptive immunotherapy approach is allogeneic hematopoietic stem cell transplantation (HSCT; Appelbaum, 2001), the only cure for several patients with high-risk hematological malignancies (Ljungman et al., 2010). The efficacy of allogeneic HSCT in patients with malignancies derives largely from the so-called ‘graft versus tumor’ (GvT) effect, an immunological response mediated by donor T lymphocytes, responsible also of the detrimental graft-versus-host-disease (GvHD; Fuchs, 2012). Gene transfer technologies, including the suicide gene approach, are promising tools to manipulate donor T-cell immunity to enforce the GvT effect, to foster functional immune reconstitution, and to prevent or control GvHD. The herpes simplex virus thymidine kinase (HSV-TK) suicide gene strategy is the most extensively tested in humans, allowing the safe infusion of a wide T-cell repertoire through the GvHD control, combined to preservation of GvT and immune reconstitution (Lupo-Stanghellini et al., 2010).

New gene-transfer-based strategies aim to enhance effector cell survival, homing, function, and safety, as well as to effectively target cancer cells by high-avidity tumor-reactive T-cell receptors (TCRs) or chimeric antigen receptors (CARs; Kalos and June, 2013; Kershaw et al., 2013). The suicide mechanism has been efficiently proposed to avoid and control the toxic effects potentially induced by these innovative cellular therapies.

Recent advances in the understanding and use of genetically engineered T-cells and monoclonal antibodies have produced unprecedented results in this emerging field. Attracted by the wide applicability of these new strategies, multiple biotech and pharmaceutical companies have consequently begun active in the clinical development of cancer immunotherapy, with the goal of offering a standardized, quality-controlled, regulatory-body-approved treatment for the integration of cell therapies to benefit patients worldwide (June et al., 2012; Maus et al., 2014). At the same time, academia is approaching a revolutionary change of point of view in its dialog with the industry, bridging a productive collaboration throughout the entire pipeline of translational medicine (Couzin-Frankel, 2013).

## Overview of Cancer Immunotherapy and Cell-Based Gene Therapy

By targeting the immune system, instead of the tumor itself, immunotherapy marks an entirely innovative way of treating cancer. Advances in the development and application of immunotherapy for cancer have been impressive in recent years, fueling optimism that this modality will soon have a meaningful impact in patient care (Mellman et al., 2011). In particular ACT, that involves the transfer of *ex vivo* expanded effector cells as a means of augmenting the antitumor immune responses, has been utilized with promising results in clinical trials (June, 2007b).

A major advantage of ACT is that the therapeutic effects can be enhanced, by isolating lymphocytes with desired effector or regulatory properties, while removing the cells that may have antagonistic effects. Direct evidence of the potency of effector T-cells to target and eradicate tumor cells was demonstrated through the clinical application of donor lymphocyte infusion (DLI) to treat leukemia after allogeneic HSCT, through the GvT effect mediated by alloreactive donor T-cells that lead to strong anti-leukemic responses in a significant portion of patients (Kolb et al., 1990; Gill and Porter, 2013).

Allogeneic HSCT is, however, complicated by the GvHD, an immune-mediated reaction against normal host epithelial tissues that is often associated with significant morbidity and mortality (Flowers et al., 2011; Jagasia et al., 2012). Despite immune suppressive drug prophylaxis, acute and chronic GvHD, arising from alloreactive donor T-cells, still represent a major complication of allogeneic HSCT (Karanth et al., 2006; Dignan et al., 2012; Arai et al., 2014; Boyiadzis et al., 2014). In most cases, non-selective lymphocyte depletion of the allograft can prevent GvHD, but lymphocyte-depleted grafts are accompanied by increased relapse rate and transplant-related mortality (TRM), mostly from infections, indicating that a fast immune recovery is essential for successful transplant outcome (Ciceri et al., 2008; Corre et al., 2010; Seggewiss and Einsele, 2010). Genetic manipulation, and suicide gene therapy in particular, may allow addressing the T-cell dilemma in allogeneic HSCT (Cohen et al., 1999).

### Rationale of Immunotherapy with Engineered T-Cells and Suicide Gene Therapy

Mature T-cells are among the most suitable cells for genetic modification. The majority of clinical approaches are based on T-cells engineered to stably express transgenes after retroviral or, more recently, lentiviral transduction (Naldini et al., 1996). Although viral-based approaches result in reasonably efficient transduction of primary T-cells, they have considerable limitations in terms of costs, DNA capacity and risks related to semi-random vector integration. In this regard, it is important to notice that insertional mutagenesis has never been observed in patients treated with genetically modified T-cells. Transduced T-cells have the potential to last the lifetime of the host and even to expand in number, therefore the clinical effect might persist, but also adverse effects attributable to permanent gene transfer may theoretically worsen over time. A potential solution is to engineer T-cells to express signaling pathways that cause the T-cell to destroy itself after a defined number of cell divisions (Friedland et al., 2009). In alternative non-viral based approaches might be used: they benefit from lower manufacturing cost and are in principle less immunogenic than viral approaches. Furthermore, such approaches are theoretically safer since they are not dependent on viral elements integrating into the host DNA. Finally transgenes will be diluted through cell division thus limiting the risks associated to cell therapy. However, the advantages of a living and persisting therapy might be largely reduced by transient transgene expression (Kalos and June, 2013). Thus, it might be advantageous to permanently express transgenes into T-cells, in association with suicide genes, that enable to selectively control their activity and life span.

A number of “suicide-gene” strategies that allow selective destruction of administered T-cells *on demand* have been developed (Hinrichs and Restifo, 2013). A suicide gene codes for a protein able to convert, at a cellular level, a non-toxic prodrug into a toxic product. In allogeneic HSCT, suicide gene modification of donor lymphocytes aims at exploiting their GvT effect, while providing a selective “switch” to GvHD (Bondanza et al., 2005). The thymidine kinase of HSV-TK is a cell cycle-dependent suicide gene, that catalyzes the generation of triphosphate ganciclovir (GCV), which is toxic to proliferating cells by inhibiting DNA chain elongation (Springer and Niculescu-Duvaz, 2000; Lamana et al., 2004; Bondanza et al., 2006). Various clinical studies with HSV-TK transduced donor lymphocyte have been performed and a Phase III multicentric, randomized clinical trial for high-risk acute leukemia is currently undergoing in the context of haploidentical HSCT.

To overcome the immunogenicity of the TK viral protein, reported in case of immune-competent patients in the autologous setting (Riddell et al., 1996) and later after non-T-cell depleted transplantation (Berger et al., 2006), alternative suicide genes have been proposed. Gene transfer of human CD20 into T-cells has been investigated as an alternative non-immunogenic suicide gene strategy, since the CD20 antigen can be employed both as a selection marker and as a target for elimination of engineered cells by administration of one of the widely used clinical-grade anti-CD20 antibodies (Serafini et al., 2004; Griffioen et al., 2009; Philip et al., 2014). Alternative suicide genes include a truncated human EGFR polypeptide (huEGFRt), which confers sensitivity to a pharmaceutical-grade anti-EGFR monoclonal antibody, cetuximab (Erbitux; Wang et al., 2011). An inducible, non-immunogenic and rapid-onset system based on a fusion protein comprised of an extracellular FK506 binding domain linked to human caspase-9 (iCasp9) signaling domains to deliver apoptotic signals in response to a small molecule-mediated dimerization has been developed and is currently being evaluated in clinical trials (Di Stasi et al., 2011). A proliferation-independent suicide gene can theoretically induce apoptosis even in non-dividing cells, including pathogen-specific precursors, determining permanent abrogation of GvHD. Nevertheless an incomplete elimination, although of ≥90% of iCasp9-modified T-cells has been reported in its clinical application (Di Stasi et al., 2011), and the residual T-cells seems to be able to re-expand, containing pathogen-specific precursors.

### Development and Implementation of TK-Suicide Gene Therapy

The rational of suicide gene therapy lays on the assumption that an intact donor T-cell repertoire, inclusive of alloreactive specificities, is required to promote a wide and protective post-transplant immune reconstitution against infections and a potential immune protection against disease recurrence. Whereas the introduction into clinical practice of less toxic chemotherapeutic agents, new antimicrobials, and more effective GvHD therapies has significantly reduced the treatment-related mortality of allogeneic HSCT over the last decades, the mortality due to disease recurrence remained largely unchanged (Gooley et al., 2010).

Suicide gene therapy enables the association of GvHD and GvT that often occurs in the same patient with different kinetics (Appelbaum, 2001). This observation suggests that a large proportion of the anti-leukemic immunological force of donor lymphocytes relies on alloreactive cells. Unfortunately, the same T-cell specificities are responsible of the detrimental GvHD. Suicide gene therapy combines the possibility to the transfer into the patients a wide T-cell repertoire, inclusive of alloreactive specificities, with a selective control of GvHD.

**FIGURE 1:**
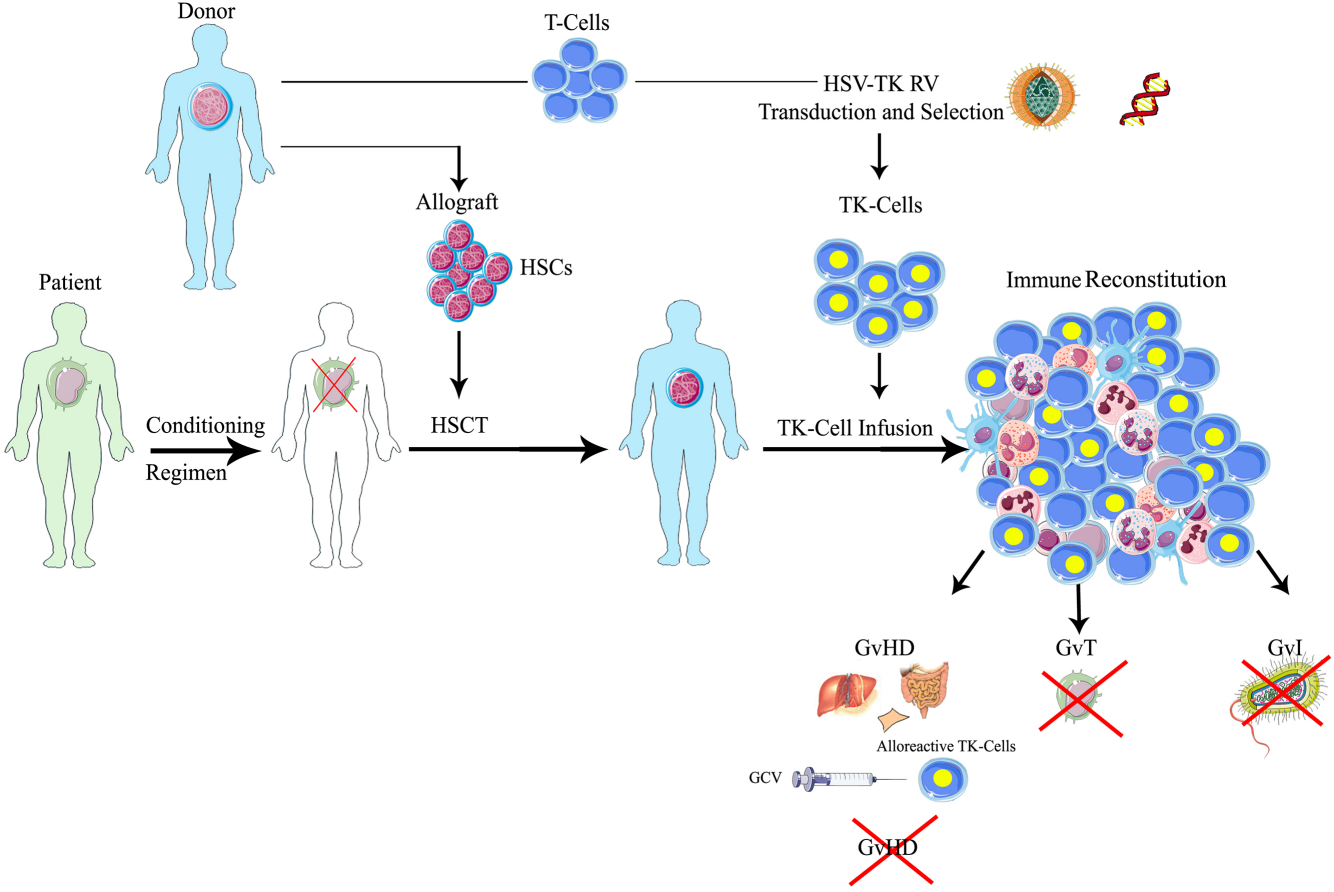
**Schematic representation of suicide gene therapy in allogeneic HSCT**. Patients affected by hematologic malignancies, after a myeloablative conditioning regimen, receive allogeneic HSCT with CD34-selected donor’s HSCs, followed by the infusion of HSV-TK gene modified donor lymphocytes. Through the *ex vivo* transfer of the HSV-TK suicide gene, T lymphocytes harvested from the same donors permanently acquire the sensitivity to a the anti-herpes drug, ganciclovir (GCV): in case of GvHD occurrence, the administration of GCV activates the suicide machinery, leading to the selective elimination of alloreactive gene-modified T-cells, while resting transduced T lymphocytes or untransduced cells are spared. Therefore, one could preserve the beneficial effects of the T-cells on engraftment, immune reconstitution and tumor control (GvT) in patients not experiencing significant GvHD. HSCT, hematopoietic stem cell transplantation; HSCs, hematopoietic stem cells; HSV-TK, herpes simplex thymidine kinase; TK, thymidine kinase; GCV, ganciclovir; GvT, graft versus tumor; GvI, graft versus infection; GvHD, graft versus host disease.

The use of HSV-TK as a suicide gene offers different levels of specificity to GvHD control: first, only transduced cells are endowed with the ability of converting the prodrug in the active form; second, only gene-modified T-cells in active proliferation are sensitive to the active drug; finally this strategy is able to control GvHD, sparing the adverse effects of post-transplant immunosuppressive prophylaxis and therapy. GCV administration is therefore meant to eliminate proliferating alloreactive T-cells, only in selected patients with GvHD, while sparing resting T lymphocytes (**Figure 1**), within a personalized therapeutic framework. GCV administration in most of the cases eliminates more than 90% of circulating TK cells (Ciceri et al., 2009). Since the frequency of alloreactive T-cells is generally low, we can speculate that they can increase their relative proportion during GvHD, while several non-alloreactive TK cells might be eliminated because of early post-transplant T-cell homeostatic proliferation. Of notice, transduced T-cells can be easily tracked in patients thanks to the presence of the ΔLNGFR (truncated low affinity receptor of nerve growth factor receptor) cell surface marker, thus providing a unique tool to study the fate of memory T-cells *in vivo* (Bonini et al., 2003).

Meanwhile, TK-cells hold an effective antileukemic activity, warranting substantial clinical benefit for a considerable proportion of patients treated with allogeneic HSCT. The objective clinical responses, inclusive of complete remissions, correlate with *in vivo* expansion of transduced cells (Ciceri et al., 2007). The long-term complete remission obtained after the infusion of TK-cells, suggests a persistent GvT activity in these patients (Oliveira et al., 2012). Moreover, the antileukemic activity of TK-cells was indirectly evidenced in the context of haploidentical HSCT by the emergence, after TK-cell infusions, of mutant variants of the original leukemia with the de-novo loss of patient-specific HLA haplotype (Vago et al., 2012b), through a newly described mechanism of relapse strongly related to the immune pressure of mismatched HSCT (Vago et al., 2009; Crucitti et al., 2014).

In addition to the GvT effect, the administration of donor TK-cells has a clinical impact in promoting immune-reconstitution (IR), thus abrogating late TRM (Marktel et al., 2003). In the TK007 phase II clinical trial, only patients with TK-cell engraftment progressed to full-term IR, whereas in the absence of circulating TK-cells, the immune recovery was dramatically slow, causing a high rate of infection-related mortality. Moreover, while TK cell infusions were necessary and sufficient to promote a rapid immune recovery, the long-term reconstituting immunity was comprised of donor-derived T lymphocytes negative for the suicide gene. Such transgene-negative T-cells were enriched for recent thymic emigrants, thus suggesting their de novo generation in the host thymus. The comparison with a cohort of patients treated with a T-cell-repleted haploidentical HSCT, suggested an active role of TK cells in supporting a thymic dependent pathway of IR, which results in the maturation and differentiation of donor hematopoietic precursors in the recipient thymus (Vago et al., 2012a). This process is remarkable in such a cohort of adult patients, usually characterized by a low thymic output. These findings show that TK cells prompt the generation of a long-lasting host-tolerant T-cell repertoire (Oliveira et al., 2012). This complete and physiological donor-derived immune system is persistently maintained in adults surviving long-term after suicide gene therapy (Oliveira et al., 2014).

The safety of this approach has been extensively confirmed by pre-clinical and clinical studies: no adverse or toxic events related to the gene transfer procedure have been reported to date. Accordingly, no genotoxic effect of integrating vectors, nor clonal dominance of gene modified T-cells has been observed (Recchia et al., 2006; Lupo Stanghellini et al., 2014).

Though highly promising, the TK-based gene therapy approach has some limitations. The tk gene is immunogenic in humans and in immunocompetent patients immunity to TK might lead to the undesired elimination of transduced cells population. The development of a CD8-mediated clearance of TK cells largely depends on the immunological status of transplanted patients at the time of TK cell infusion (Traversari et al., 2007). The immunogenicity of this viral protein is not limiting its application in haploidentical HSCT, where the recipient is profoundly immunosuppressed (Riddell et al., 1996) and no immune response against TK has been observed (Ciceri et al., 2009). An additional limitation derives from the nature of GCV which is a useful drug to treat cytomegalovirus (CMV) reactivation, often occurring in immunocompromised patients; in these patients, the administration of GCV to treat CMV would produce unwanted TK-cell killing. Finally, TK/GCV may have limited ability to actually kill cell populations, particularly those that are post-mitotic. While in some clinical conditions, the selective elimination of proliferating alloreactive T-cells, selectively in patients with GvHD, while sparing resting T lymphocytes, allow a ‘personalized’ modulation of alloreactivity and preserve pathogen-specific precursors, in other settings, a suicide gene/prodrug system independent of the cell cycle could be advantageous (Bonini et al., 2011). A suicide gene system independent of the cell cycle could be favorable to completely eradicate all cell populations, including post-mitotic or resting cells, such as less-proliferating alloreactive cells (i.e., memory T-cells) or slowly dividing cells. Of course, a suicide gene strategy should be adapted for each specific application, and the final choice may have to take into consideration the advantages and disadvantages peculiar of each approach.

### Clinical Application of the TK Suicide Gene in the Context of Allogeneic HSCT

The exploitation of suicide genes in the context of allogeneic HSCT to control T-cell reactivity represents one of the most important demonstration of the feasibility, safety, and clinical relevance of gene therapy (Cieri et al., 2014). Up to the present, TK cells have been infused within several clinical trials (**Table 1**) in 148 patients after HLA-identical and haploidentical HSCT (Bonini et al., 1997; Munshi et al., 1997; Champlin et al., 1999; Tiberghien et al., 2001; Burt et al., 2003; Fehse et al., 2004; Onodera et al., 2006; Ciceri et al., 2007, 2009). The cumulative follow-up of patients enrolled in different clinical trial at San Raffaele Scientific Institute, treated with TK cells, is 289 person/year; twelve out of them are alive and well at more than 5 years from HSCT (unpublished data). Every case of acute and chronic GvHD occurring after the infusion of TK cells has been controlled, confirming the efficacy of the suicide gene/prodrug system in controlling alloreactivity (Bonini et al., 2011). *In vivo* depletion of TK cells in the setting of chronic GvHD is crucially dependent by a stable HSV-TK expression for several months after HSCT (Oliveira et al., 2014). Nevertheless the incidence of chronic GvHD was low in HSV-TK studies, probably because of the thymic dependent pathway of long-term immune reconstitution (Vago et al., 2012a).

**Table 1:** Clinical trials of TK-suicide gene therapy in allogeneic HSCT.

Clinical application	Vector (suicide gene/marker gene)	Days of culture	N° of treated patients	Clinical response (n° of patients)	Incidence of GvHD n° pts	Complete response of GvHD to GCV	Immunity against HSV-TK	Reference
To treat disease relapse occurring after HLA-identical allogeneic HSCT	RV (HSV-TK/ΔLNGFr) RV (HSV-TK/NeoR) RV (HSV-TK/NeoR) RV (HSV-TK/NeoR) RV (HSV-TK/ΔLNGFr)	14 Ne Ne 24–48 9–11	23 23 3 9 5	11^a^ 6^a^ 1^a^ 2^a^ 4^a^	4 0 1 1 2	3/3^b^ Ne Ne 1/1 2/2	9/23^f^ Ne Ne Ne Ne	Bonini et al. (1997), Ciceri et al. (2007) Champlin et al. (1999) Munshi et al. (1997) Burt et al. (2003) Onodera (2008)
Day 0 in TCD allogeneic HSCT	RV (HSV-TK/NeoR) RV (HSV-TK/NeoR)	12 –	12 3	4^a^ 1^a^	5 1	5/5^c^ 1/1	4/12 Ne	Tiberghien et al. (2001) Fehse et al. (2004)
Day 60 in TCD allogeneic HSCT	RV (HSV-TK/ΔLNGFr)	10	9	7^a^	1	1/1	1/9	Borchers et al. (2011)
Day 42 in TCD haploidentical HSCT	RV (HSV-TK/ΔLNGFr) RV (HSV-TK/ΔLNGFr) RV (TKmut2/ΔLNGFr)	14 10 10	8 28 4	3^d^ 22^d^ 4^d^	1 11 0	1/1 10/10^e^ Ne	0/8^f^ 0/28^f^ Ne	Bonini et al. (2007) Ciceri et al. (2009)
**Total**			127	61	27	24/24	14/127	

*The table includes clinical data about patients treated with TK-suicide gene strategy in the context of allogeneic HSCT. HSCT, hematopoietic stem cell transplantation; GCV, ganciclovir; Ne, not evaluable; HSV-TK, herpes simplex thymidine kinase; TCD, T-cell depleted; GvHD, graft versus host disease. ^a^Clinical outcome is measured as clinical response of the malignant disease. ^b^One patient with GvHD achieved complete response (CR) after GCV administration and immunosuppressive drugs. ^c^One patient with GvHD achieved CR after administration of GCV and steroids. ^d^Clinical outcome is measured in terms of T-cell immune reconstitution (evaluated as more than 100 circulating CD3+ T lymphocytes/μl). ^e^Four patient with GvHD achieved CR after administration of GCV and short course low dose steroids, two patients with GvHD achieved CR after GCV administration and immunosuppressive drugs. ^f^Immunity against HSV-TK was monitored by the appearance of TK–specific CD8+ effectors, leading to undesired elimination of a transduced cell population (Traversari et al., 2007).*

We initially reported the use of donor T-cells expressing TK gene to treat Epstein–Barr virus-induced B lymphoproliferative disorders after allogeneic HSCT and leukemia relapse in the context of HLA-identical HSCT (Bonini et al., 1997, 2003). The activation of the suicide gene machinery granted successful control of acute or chronic GvHD in all the patients who required GCV administration. Moreover, a considerable portion of the patients experienced a substantial benefit from gene manipulated DLIs: the *in vivo* expansion of TK cells directly correlated with the achievement of a partial or complete remission from disease (Ciceri et al., 2007).

Based on these encouraging results, the TK approach was then translated into the more challenging mis-matched family haploidentical HSCT, historically limited by a high rate of late TRM and relapse incidence, associated with a delayed IR secondary to the procedures for severe GvHD prevention and treatment. Different strategies have been developed to modulate, abrogate or control T cell-mediated alloreactivity, with the purpose of reducing the risk of GvHD while preserving post-transplant IR and GvT activity in haploidentical HSCT. T-cell depletion (TCD), achieved through CD34+ positive selection of the graft in the absence of post-grafting immunosuppression, is associated to low rates of acute GvHD and absence of chronic GvHD, but is marked by a rate of non-relapse mortality (NRM) of ~40%, largely due to infections and a cumulative incidence of relapse of 51% for patients transplanted in relapse (Aversa et al., 2005; Ciceri et al., 2008). The group of Perugia has recently reported results of a protocol based on the infusion of donor Tregs following a TCD haploidentical transplant to further reduce the risk of GvHD (Di Ianni et al., 2011). Based on the evidence that alloreactivity appears to cluster with specific T-cell subsets (such as CD8 or naïve T-cells, or αβ T-cells), the infusion of donor T-cells depleted of selected T-cell subsets has been also tested in clinical trials (Bertaina et al., 2014). In the last years, haploidentical HSCT has gained considerable attention worldwide, due to the development of new promising tools to prevent GvHD, such as the post-transplant administration of high-dose cyclophosphamide and the use of granulocyte-colony stimulating factor-primed bone marrow (Luznik et al., 2012; Di Bartolomeo et al., 2013; Raiola et al., 2013); based on these two innovations, the number of transplants performed using unmanipulated haploidentical grafts has grown. A sirolimus-based GvHD prophylaxis has also been adopted to allow the infusion of unmanipulated peripheral blood stem cells (PBSCs) grafts from haploidentical donors, and proved able to promote a rapid immune reconstitution skewed toward T-regulatory cells (Peccatori et al., 2014). Despite all these efforts, acute and chronic GvHD still represent crucial complications of haploidentical HSCT.

In the heterogeneus panorama of allogeneic transplants, the TCD haploidentical HSCT still represents an ideal platform to develop and validate innovative adoptive immunotherapy approaches: the rapid and robust hematopoietic reconstitution, associated to a profound lymphodepletion and to the absence of immunosuppressive drugs, represent favorable grounds for the engraftment and expansion of adoptively transferred T-cells.

In the TK007 multicentric phase I-II clinical trial, 50 adult patients were enrolled to receive a TCD haploidentical HSCT followed by the infusion of TK cells, for high-risk hematologic malignancies. Again TK cells proved safe, with no documented adverse event related to the genetic modification. In this study, 28 adult patients were treated with multiple infusions of TK cells starting 1 month after haploidentical HSCT, in the absence of pharmacologic prophylaxis for GvHD; 22 patients obtained immune reconstitution at a median of 75 days (range 34–127) from HSCT and 23 days (13–42) from TK-cell infusion. Twelve patients developed acute GvHD, and one developed chronic GvHD. Direct association of TK-cells and GvHD was confirmed by vector-encoded protein immunostaining of lymphocytes infiltrating affected lesions. In all cases the TK/GCV machinery proved effective, leading to a rapid and complete resolution of all clinical symptoms and providing a long-term immunosuppressive therapy free survival, in absence of GvHD related deaths or long-term complications (Lupo Stanghellini et al., 2014). Beside the efficient control of donor T-cell alloreactivity, the infusion of TK cells granted a rapid and wide immune reconstitution, which was instrumental for the abrogation of the late TRM, reported for TCD haploidentical HSCT. In immune reconstituted patients, progressive normalization of antiviral responses was associated with a decline in the number of infectious events, while patients who failed immune reconstitution continued to have frequent infectious complications. At 3 years, intention to treat NRM was ≈40%, with all events occurring in the first 6 months after HSCT. For patients with primary acute leukemia, transplanted in complete remission, relapse mortality at 3 years was 19% (Ciceri et al., 2009). All patients in complete remission 3 years after transplant remained so in the following years (longest follow-up 11 years; Oliveira et al., 2012).

The HSV-TK strategy is currently under evaluation in a phase III clinical trial in patients undergoing haploidentical HSCT for high-risk acute leukemia. In this pivotal, ongoing phase III trial (TK008, NCT00914628), up to 4 monthly infusions of TK cells are given at 1x10^7^ for kg of patient body weight, starting 21–49 days after TCD haploidentical HSCT in the experimental arm. Control arm consists of either TCD or post-transplant cyclophosphamide haploidentical HSCT, at physician discretion. So far, 34 patients have been enrolled from eight EU and US sites. Preliminary results of this ongoing trial (median follow-up is 1.2 years) confirm the potential clinical benefit of T-cell gene transfer technology integrated with TCD haploidentical HSCT, and highlight the role of early IR as surrogate endpoint for survival outcomes and the dose-related antileukemic effects of TK (Bonini et al., 2014).

Herpes simplex virus thymidine kinase suicide gene strategy, employed as cytotoxic therapy, may also improve the outcomes of solid tumors, especially as adjuvant to local therapies (Nanda et al., 2001; Li et al., 2007; Xu et al., 2009; Aguilar et al., 2015).

The efficacy of the suicide gene approach in controlling the adverse events associated to DLIs open up to the opportunity to increase the safety profile of additional T-cell therapy approaches, such as that based on the expression of tumor redirected T-cells, obtained through TCR gene transfer or CAR based gene therapy.

### Adoptive T-Cell Therapy and TCR Gene Transfer

ACT is based on the possibility to isolate tumor-specific T-cells from the patient’s peripheral blood, or from tumor samples (TILs; Rosenberg, 2012). TIL-based approaches derive from the concept that T lymphocytes in biopsy specimens are enriched for anti-tumor reactivity but have become functionally anergized, and that *ex vivo* culture of these cells results in their re-activation. However, their application is currently limited by a number of factors: for several cancers tumor specimens are not readily available, even when available TILs are reproducibly detectable only in a minority of cancer types, expansion protocols remain relatively labor-intensive, expensive, and difficult to standardize (Ruella and Kalos, 2014). Furthermore, prolonged T-cell culture required to expand rare tumor-specific T-cells often results in their functional exhaustion.

Thus, several challenges need to be faced for a successful adoptive cancer immunotherapy: the extent of T-cell activation, the T-cell differentiation phenotype and most importantly the T-cell specificity of the cellular product represent variables able to significantly affect the efficacy and safety of the cell therapy approach (Kershaw et al., 2014). Indeed for an effective adoptive immunotherapy, the choice of the tumor associated antigen (TAA) or epitope to be targeted is critical. An ideal tumor antigen should be highly specific for cancer cells, originated from a founder mutation, possibly required for the oncogenic phenotype and should be widely expressed in different patients and possibly shared by different tumor types (Restifo et al., 2012). In Cheever et al. (2009), the National Cancer Institute published a list of ‘ideal’ cancer antigens, based on several features (immunogenicity, oncogenicity, specificity, frequency) with the aim of promoting translational research in the field of adoptive immunotherapy.

The fundamental premise behind genetic retargeting of T-cells is the fact that the endogenous potent tumor-specific T-cell repertoire has been compromised as a consequence of central and peripheral tolerance. Indeed, comparative analyses have demonstrated that TCRs against tumors have substantially lower antigen affinity (~0.5 logs) compared with TCRs directed against virus-derived antigens, providing at least partial explanation for the lack of clinical efficacy of approaches directed to triggering the self-antigen-reactive T-cell repertoire (Aleksic et al., 2012). Genetic engineering of circulating autologous lymphocytes from cancer patients by using genes that encode receptors capable of recognizing cancer antigens is used to generate high-avidity T-cells with specificity for tumors. These high-avidity T-cells can be equipped with conventional TCRs expressing a heterodimeric αβ receptor, which recognize processed antigenic peptides presented by major histocompatibility complex (MHC) proteins or with CARs, which recognize TAA through single chain variable fragments that are isolated from antigen-specific mAbs.

Gene encoding TCRs, made up of α- and β-chains, can be obtained from tumor-specific T-cells, which can naturally occur in humans, and cloned into a transfer vector, more often a retroviral or lentiviral vector, which can then be used to transduce mature T lymphocytes. TCR affinity can be increased through several approaches, such as the introduction of amino acid substitutions into the CDR regions of α and β chains, especially into the peptide-binding CDR3 regions, or by the modulation of the TCR glycosylation (Li et al., 2005; Chervin et al., 2008; Robbins et al., 2008; Kuball et al., 2009; Schmitt et al., 2013). The genetic transfer of tumor-specific TCR α and β can allow the generation of high number of antigen-specific T-cells from virtually every patient. This approach bypasses the need to isolate the tumor-specific effector cells from each patient. TCR-engineered T-cells secrete immunostimulatory cytokines, exert antigen-specific cytotoxicity upon encounter with antigen-positive tumor cells and expand in response to this antigenic stimulation (Johnson et al., 2009). This approach has been applied with a variety of antigens including MART-1 and gp100 (melanoma); NY-ESO-1 (epithelial tumor and sarcomas); CEA (colorectal cancer); and 2G-1 (renal cell carcinoma; Johnson et al., 2009; Robbins et al., 2011). To completely and permanently abrogate the expression of the endogenous TCR and the risk of mispairing between the endogenous and the tumor-specific TCR chains, a novel approach, named TCR gene editing, has been recently developed (Provasi et al., 2012). This approach aims for the first time to substitute, instead of adding, the T-cell specificity, thus increasing the efficacy and safety profile of the cellular product, and permitting a safe use also of allogeneic cells, by abrogating the risk of GvHD. However, even this approach can be complicated by potential “on-target, off-organ” adverse effects caused by recognition of low-level expression of the targeted tumor-associated antigen on healthy tissues, which can be recognized by high-affinity TCRs (Johnson et al., 2009). The major challenge for immunotherapy using gene-modified T-cells remains the identification of antigens that can be targeted to destroy the cancer without causing toxicity to normal tissues. In a recent clinical trial using T-cells engineered to express an affinity enhanced TCRs specific for a MAGE-A3 derived epitope, HLA-A1 restricted in patients with multiple myeloma and melanoma, the first two treated patients developed cardiogenic shock and died within a few days after T-cell infusion, due to T-cell cross-recognition of a titin-derived peptide, expressed by cardiomyocites (Cameron et al., 2013; Linette et al., 2013). The cross-recognition could not have been predicted from preclinical studies, and indicates the need to increase the safety profile of new cellular products.

Recently, the increasing use of high throughput screening of cancer genomes that allow to identify a large numbers of novel TAA, including mutated antigens, has opened up a new therapeutic window for a highly effective, ultrapersonalized adoptive T-cell therapy approach (Rosenberg and Queitsch, 2014). The wider will be the range of tumor antigens that can be targeted by TILs and genetically modified cells, the highest will inevitably be the risk of toxicity, again suggesting the need for selective approaches, such a implementation of suicide genes in transfer vectors, to eliminate the adoptively transferred cells in case of severe adverse events.

### CAR-Based Gene Therapy

The use of CARs, that recognize non-MHC-restricted structures on the surface of target cells, is highly attractive for several reasons: first it allows to overcome HLA-restriction, thus permitting to use the same CAR for several patients, second, because tumors can frequently fail to present tumor antigens to T-cells through the down-regulation of MHC expression (Curran et al., 2012), and such mechanism would not affect CAR efficacy. CAR engineered T-cells combine the antigen specificity and high avidity of antibodies and the cytotoxic properties of T lymphocytes. CARs are generated by fusing the antigen-binding motif of a monoclonal antibody (mAb) with the signal transduction machinery of the TCR. The genetic modification of T lymphocytes with chimeric receptors specific for TAA allows for their redirection toward tumor cells. In contrast to antibodies, CAR-modified T-cells can specifically traffic to tumor sites and persist, at least for some time, as memory cells *in vivo*. The most advanced CAR-based approach, redirects T-cells against CD19, a differentiation molecule of the B cell lineage. More than 200 patients affected by acute lymphoid leukemia, chronic lymphocytic leukemia, and non-hodgkin lymphoma have been treated to date with clinical responses rates reaching more than 90% (Porter et al., 2011; Grupp et al., 2013; Davila et al., 2014; Maude et al., 2014). Most responses are complete clinical responses and were achieved in highly pretreated patients, who had often failed transplantation and treatment with anti-CD19 antibodies. This exciting clinical experience is, however, limited by some degree of toxicities (Brentjens et al., 2010). The “on-tumor toxicity” is due to excessive cytokine release, associated to the use of second-generation CD19-CAR T-cells. The cytokine release syndrome is acute and usually reversible, associates with massive elevation of plasma IL-6 and can be treated with anti-IL6r antibodies. Severe forms, usually associated with high tumor burden, lead to hypotension and respiratory distress. These observations prompted to draw some clinical recommendations, including implementing careful dose-escalation plans and co-expressing a suicide gene for switching-off unpredicted or controlling long-term toxicities (Ertl et al., 2011). Currently, there are a number of active and recruiting phase I/II clinical trials aiming at the demonstration of the safety and the efficacy of CARs both in the US and in Europe (Kohn et al., 2011; Ramos and Dotti, 2011). The majority of clinical trials are focused on lineage marker-specific CARs, that is CARs specific for molecules selectively expressed by the malignant counterpart of normal blood cells (Casucci et al., 2015). In addition to the successful CD19-specific CARs, a number of novel oncoantigen-specific CARs, that is CARs specific for antigens whose expression is somewhat linked to the malignant phenotype, have been described and have entered the clinical arena for some types of liquid and solid tumors (Casucci et al., 2015). Novel CAR-based approaches have been recently designed to increase the efficacy and safety profile of CAR-based therapy. We developed a CAR specific for CD44v6, a CD44 variant highly expressed by several tumors and associated to chemoresistance. To overcome chronic toxicity that might result from the *in vivo* ablation of CD44v6 expressing monocytes, we implemented the suicide gene approach in the vector (Casucci et al., 2013). A recent study show that huEGFRt represents a highly efficient transgene-encoded cell surface polypeptide for selection, *in vivo* tracking, and ablation of CAR-engineered T-cells. (Wang et al., 2011) A novel construct also incorporates the IL-15 gene and iCasp9-based suicide gene in T lymphocytes expressing a CAR targeting the CD19 antigen, to safely increase the anti-lymphoma/leukemia effects of CAR in patients with B-cell malignancies (Hoyos et al., 2010).

## Conclusion and Future Perspectives

Cancer cellular therapy is an emerging and promising field, and a built-in suicide mechanism has been successfully combined to improve the safety of novel approaches. Thanks to a 20-years consolidated clinical experience, confirming its efficacy and flexibility, the application of TK-suicide gene strategy can be easily extended to different medical needs.

A fundamental area is the use of suicide gene therapy for DLI infusions after HSCT, to operate a meaningful dissociation between the GvT effect and GvHD.

Donor lymphocyte infusion can produce lasting remissions in patients with relapsed chronic myeloid leukemia, but are less effective in other hematological diseases. Combination of antitumor agents with DLI or the use of donor T-cells encoding a CAR targeting a tumor antigen might be the solution to further increase the GvT effect of DLI. Chemotherapy-induced lymphodepletion (i.e., cyclophosphamide and fludarabine) before DLI has been shown not only to enhance activation of donor lymphocytes but also to cause significantly more severe GvHD than DLI alone, thus limiting its application (Schmid et al., 2007). In the search for novel treatment strategies, azacytidine in addition to DLI has been employed to improve the survival of relapsed acute myeloid leukemia or myelodysplasia occurring after allogeneic HSCT (Czibere et al., 2010), while bendamustine followed by escalating doses of DLI appears to be effective as salvage treatment in hodgkin lymphoma relapsing after allogeneic HSCT (Sala et al., 2014). However, acute and chronic GvHD are reported in a significative proportion of patients. To safely balance the toxic versus beneficial effects of activated donor lymphocytes, the infusion of donor T-cells engineered to carry a suicide gene has been evaluated for treating patients with aggressive hematologic malignancies (Maury et al., 2014). Strategies that combine ACT with the use of agents that impact tumor biology such as demethylating agents, tumor signaling, metabolic pathway and cell cycle inhibitors, will need to be better investigated in the next future. Finally, an attractive option is the combination of ACT with T-cell engaging bispecific antibodies, which target T-cells and tumor cells (Topp et al., 2014).

Since suicide gene therapy for HSCT has demonstrated the potential to safely balance GvT and GvHD, it is reasonable to think that the implementation of a suicide gene in CAR-redirected T-cells may help mitigating their risks, while preserving their therapeutic effects (Casucci et al., 2013). Although TCR- and CAR-redirected T-cells are in general well tolerated (**Table 2**), their broader use requires having solid strategies to treat or, better, prevent on-target, off-tumor effects and cytokine storms. Applying suicide gene modification to TCR- and CAR-redirected T-cells may therefore greatly increase their safety profile and facilitate their clinical development (Hoyos et al., 2010; van Loenen et al., 2013).

**Table 2:** TCR- and CAR-redirected T-cells.

	TCR	CAR
Targetable Ag:	
✔Intracellular Ag	+	−
✔Surface Ag	+	+
✔Lipid Ag	+/−*	+
Ag processing required	+	−
HLA restriction	+/−*	−
Persistence	++	+
Indications for suicide gene implementation:	
✔cytokine storm	+	++
✔on target/off tumor recognition	+/−	+/−
✔off target/off tumor recognition ++-	+**	−

The table describes the different characteristics of TCR- and CAR-redirected T-cells. TCR, tumor-reactive T-cell receptor; CAR, chimeric antigen receptor; Ag, antigens; HLA, human leukocyte antigen. *Some TCRs have been shown to recognize tumor-associated lipids restricted on CD1 molecules (Lepore et al., 2014), **potentially abrogated by TCR gene editing.

Moreover, the implementation of a pre-established suicide system represents an efficient method to control survival and avoid systemic toxicity of genetically modified mesenchymal stem cells (MSCs) and hematopoietic stem cells (HSCs; De Palma et al., 2003; Bourgine et al., 2014). MSCs, ideally suitable for the immunomodulatory properties and in regenerative medicine, are also promising vehicle for targeted cancer gene therapy because of their potential tumor tropism (Choi et al., 2012; Lee et al., 2013). Innovative approaches are thus revisiting the mythic trojan horse concept to carry therapeutic nucleic acids to pathologic tumor site (Collet et al., 2013). More consolidated is the use of HSCs for the cell and gene therapy of several inherited and acquired diseases. HSC therapy using retroviral or lentiviral vectors is a promising approach to provide life-long correction of genetic defects, successfully applied to severe combined immunodeficiency-X1, ADA-SCID, adrenoleukodystrophy, β-thalassemia, Wiskott–Aldrich-syndrome, and metachromatic leukodystrophy (Larochelle et al., 1996; Cavazzana-Calvo et al., 2000; Aiuti et al., 2002; Frittoli et al., 2011; Aiuti et al., 2013; Biffi et al., 2013). However, genetic modification with viral vectors in general and stable integration of the therapeutic gene into the host cell genome bear safety concerns; in particular, insertional mutagenesis by enhancer mediated dysregulation of neighboring genes or aberrant splicing is still a major issue (Kohn et al., 2013). To alleviate the risks related to the persistence and potential genotoxicity of such cellular therapies, the inclusion of a suicide gene to ablate gene-modified cells has been undertaken in some cases (Gschweng et al., 2014).

Furthermore, new and emerging area could benefit from the suicide gene approach. During the last decade considerable clinical progress has been achieved in allogeneic HSCT for hematological disorders, generating interest in extending its application to non-malignant disorders (i.e., thalassemia, aplastic anemia; Bernardo et al., 2012). Allogeneic HSCT can provide long-term disease control also in immune-mediated neurological diseases, and a durable clinical remission was reported after allogeneic HSCT in two patients suffering from severe forms of neuromyelitis optica, suggesting HSCT as a treatment option for patients with aggressive and refractory forms of the disease (Greco et al., 2014). In this peculiar setting, the incorporation of an inducible suicide gene and its pharmacologic activation in case of GvHD, to efficiently eliminate gene-modified T-cells, could potentially increase the safety and extend the application of allogeneic HSCT also in non-malignant diseases.

In conclusion, advances in the understanding of the T-cell biology and T-cell engineering have provided multiple novel adoptive transfer strategies to maximize cure, that are now poised for translation into clinical trials, safely and easily managing possible toxicities with the suicide gene strategy.

## Author Contributions

All the authors contributed to conception, acquisition, and analysis of data, participated in the manuscript draft preparation, revision and approved and revised the final version.

## Conflict of Interest Statement

Professor Claudio Bordignon is an employee of MolMed S.p.A., Milano, Italy. Doctor Chiara Bonini receives a research grant from Molmed S.p.A., and has been a consultant of Molmed S.p.A. None of the other Authors have any conflict of interests to disclose.
